# Datasets from a vapor diffusion mineral precipitation protocol for *Dictyostelium* stalks

**DOI:** 10.1016/j.dib.2016.04.019

**Published:** 2016-04-13

**Authors:** Magdalena Eder, Christina Muth, Ingrid M. Weiss

**Affiliations:** INM – Leibniz Institute for New Materials, Campus D2.2, 66123 Saarbruecken, Germany

## Abstract

Datasets from a slow carbonate vapor diffusion and mineral precipitation protocol for *Dictyostelium* ECM and cellulose stalks show examples for composite materials obtained by an in vitro approach, which differs substantially from the in vivo approach reported in The Journal of Structural Biology, doi: 10.1016/j.jsb.2016.03.015 [1]. Methods for obtaining the datasets include bright field transmitted light microscopy, fluorescence microscopy, LC-PolScope birefringence microscopy, variable pressure scanning electron microscopy (VP-SEM/ESEM), and Raman imaging spectroscopy.

**Specifications Table**

**Table:** 

Subject area	*Materials Science, Chemistry, Biology*
More specific subject area	*Biomineralization*
Type of data	*Images (microscopy, spectroscopy), text file, figures*
How data was acquired	*Inverted Light Microscope Cell Observer Z1 (Zeiss, Göttingen, Germany), LC-PolScope/Abrio System (CRI, LOT-Oriel, Darmstadt, Germany), VP-SEM Quanta FEI 400F (FEI, Eindhoven, Netherlands), Confocal Raman spectrometer Aramis Labram (Horiba Jobin Yvon, Bensheim, Germany)*
Data format	*Raw, analyzed (overlay images)*
Experimental factors	*Native biological samples (Dictyostelium ECM) were subjected to slow precipitation of calcium carbonate in aqueous Ca*^*2+*^*solution with ammonium carbonate in the vapor phase*
Experimental features	*Specimens were investigated in wet or dry conditions, without any further modification*
Data source location	*n/a*
Data accessibility	*Data is with this article.*

## Value of the data

• Data could serve for comparison with minerals precipitated without organic matrix.
• Data could serve for comparison with calcifying ECM of other cell types.
• Data could serve for comparison with *Dictyostelium* ECM, where minerals were precipitated using alternative methods.

## 1. Data

Datasets show fluorescence light microscopy, LC-PolScope/Abrio imaging, scanning electron microscopy (VP-SEM), and confocal Raman imaging spectrometry data of composite materials obtained from *Dictyostelium* extracellular matrix (ECM). Different parts of native and genetically modified ECM were subjected to slow precipitation of calcium carbonate in aqueous Ca^2+^solution with ammonium carbonate in the vapor phase. Datasets of specimens modified with different protein derivatives, either from egg shell (OC-17) or from mollusc shells (n16N, perlucin) investigated in wet or dry conditions and without any further modification are presented*.*

## 2. Experimental design, materials and methods

### 2.1. Cell lines

Synthetic sequences of n16N, OC-17 and perlucin were cloned into modified Gateway expression systems for *Dictyostelium* Ax3-Orf+ under the control of the [ecmB] promoter [1]. The heterologous proteins were expressed with a C-terminal GFP domain for detection, and an N-terminal *Dictyostelium* signal peptide to facilitate their export into the extracellular matrix (ECM) which is formed during the multicellular stages of the *Dictyostelium* life cycle.

### 2.2. In vitro mineralization experiments

*Dictyostelium* ECM mineral composites were produced under slow ammonium carbonate vapor diffusion adapted from in vitro precipitation assays [2], [3], [4], [5]. The 2–3 days old organic stalks of differentiated *Dictyostelium* cell lines were collected using tweezers and spore heads were gently removed. The stalks were immersed in 25 μl of 10 mM CaCl_2_, pH 6.0±0.1 (or in the presence of 3 mM glycylglycine, pH 8.5–8.7) in disposable microscopy slides (μ-slide, #81821, ibidi, Martinsried, Germany). The μ-slide was covered with gently perforated aluminum foil. Slides were incubated in humid atmosphere 12 h to 2 days in a closed incubator at room temperature containing 0.5 g of (NH_4_)_2_CO_3_ until the pH increased to 8.7±0.2. For drying the specimens, the Ca^2+^containing salt solution with suspended organic stalks was quickly transferred onto nitrocellulose filters (0.2 μm pore diameter, Millipore) on top of a filter holder mounted on a vacuum pump, rinsed twice with 100 μl deionized water and immediately air dried under vacuum.

### 2.3. Transmitted light microscopy and fluorescence microscopy

**Fig. 1:**
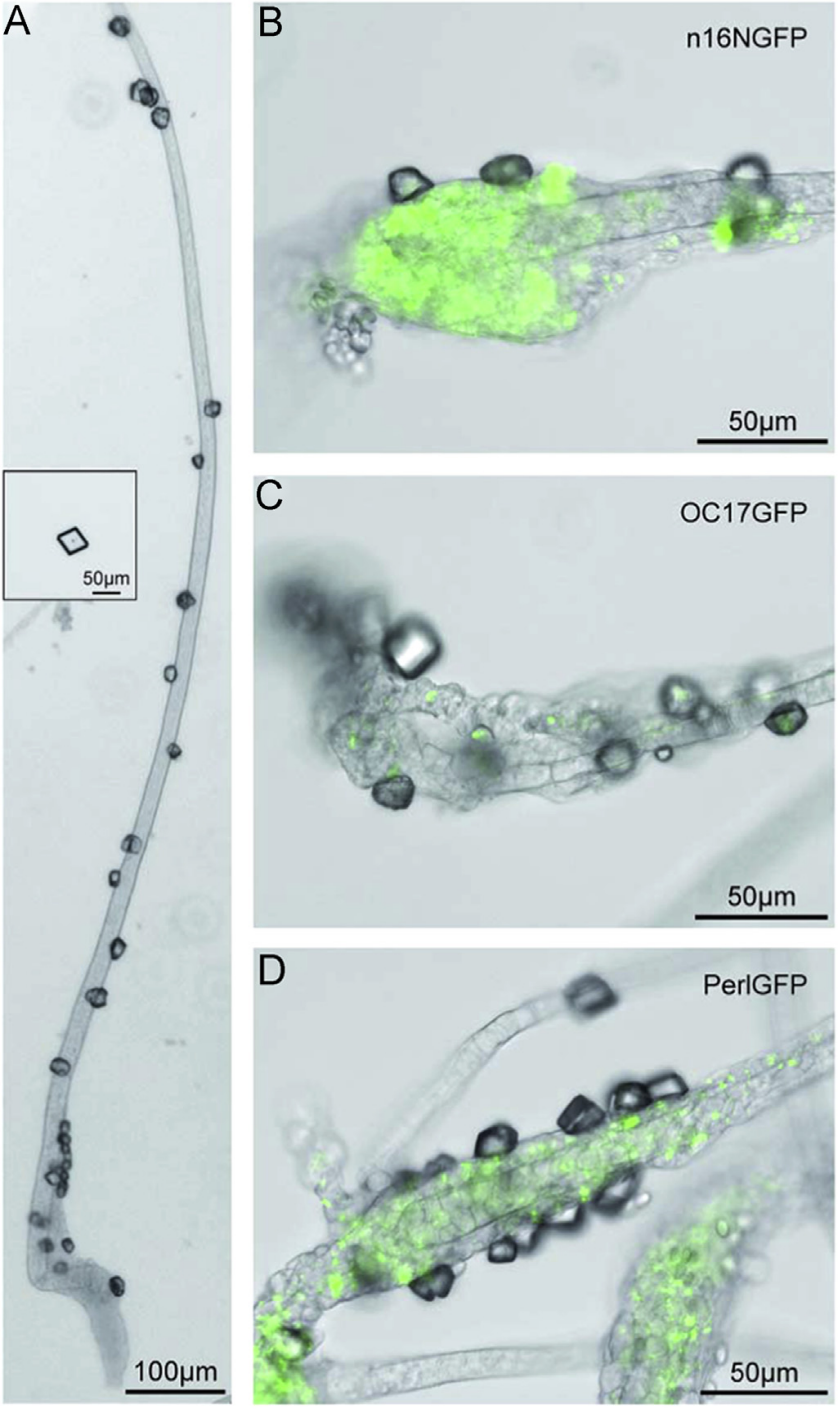
Calcium carbonate precipitates associated with *D. discoideum* Ax3-Orf+ stalks as obtained by the ammonium carbonate vapor diffusion protocol (a), and calcite crystal grown in precursor solution under identical conditions without organic additives as a control experiment (a, insert). Ax3-Orf+ (a) and transformed cell lines AX3_n16NGFP (b), AX3_OC17GFP (c), and AX3_PerlGFP (d) are shown in overlay microscopy images taken in transmitted light and fluorescence imaging modes.

Specimens were investigated using an inverted light microscope Cell Observer Z1 equipped with a mercury arc lamp and a Colibri LED fluorescence excitation system and the objectives A-Plan 10×/0.25Ph1 and LD Plan Neofluar 40×/0.6Korr Ph2 (Zeiss, Göttingen, Germany). Electronic images were recorded with an Axiocam MRm Camera and Axiovision 4.8 software. Transmitted light microscopy images were taken at automatic exposure times. Fluorescence microscopy images were taken at a fixed exposure time of 10 s using a band pass filter (excitation 470/40, emission 525/50) at 50% Colibri LED intensity (Fig. 1).

### 2.4. LC-PolScope quantitative birefringence microscopy

**Fig. 2:**
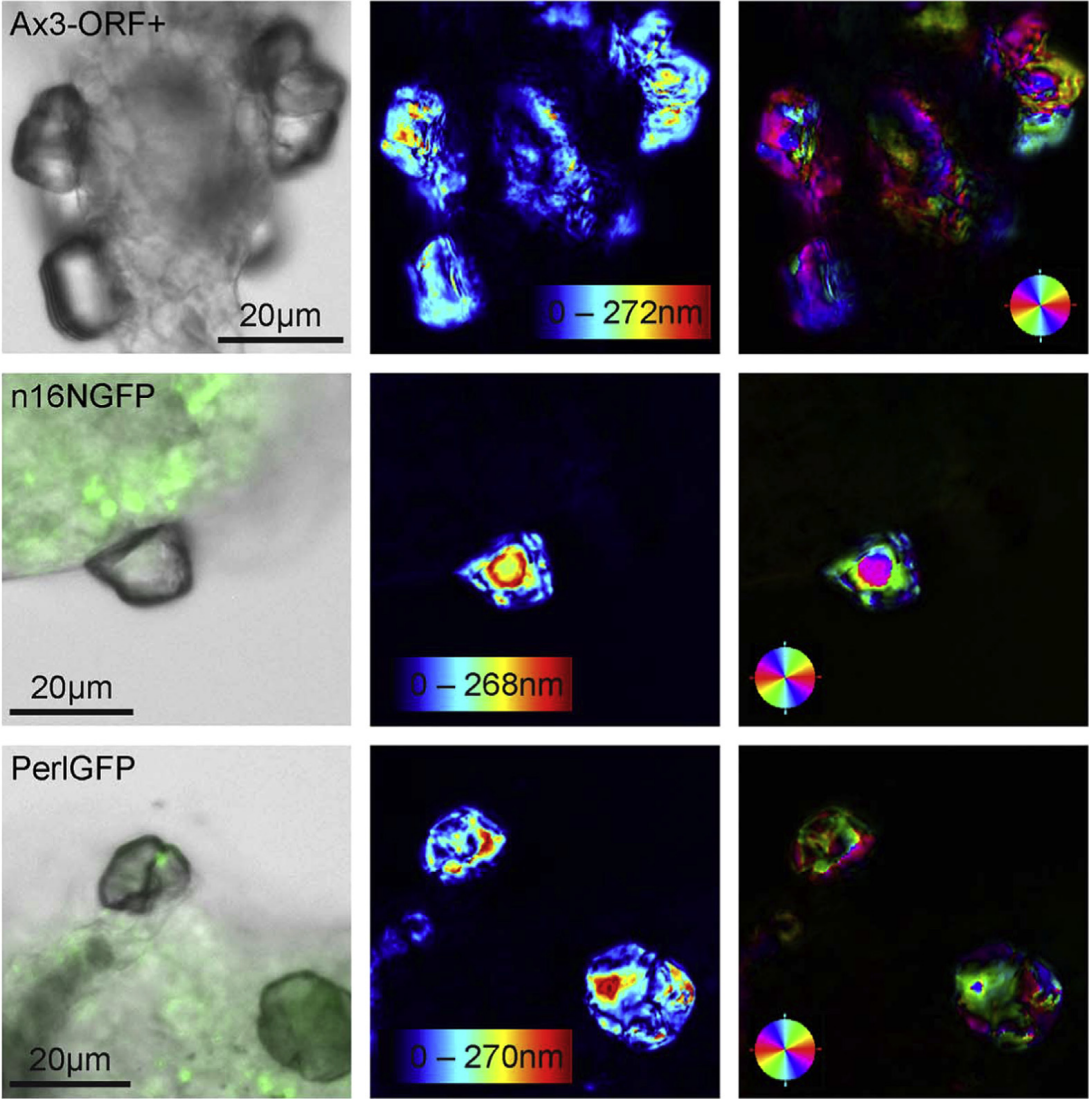
Calcium carbonate precipitates associated with *D. discoideum* Ax3-Orf+ stalks as obtained by the ammonium carbonate vapor diffusion protocol. Region 1, the so-called basal region, of Ax3-Orf+ (top row), and of AX3_n16NGFP (central row) and AX3_PerlGFP (bottom row) in transmitted light and fluorescence overlay microscopy images (left column), and LC-PolScope images analyzed in the retardance mode (center) and in the orientation mode (right column), with slow optical axis indicated in red 0°/180°, light blue 90°/270°.

**Fig. 3:**
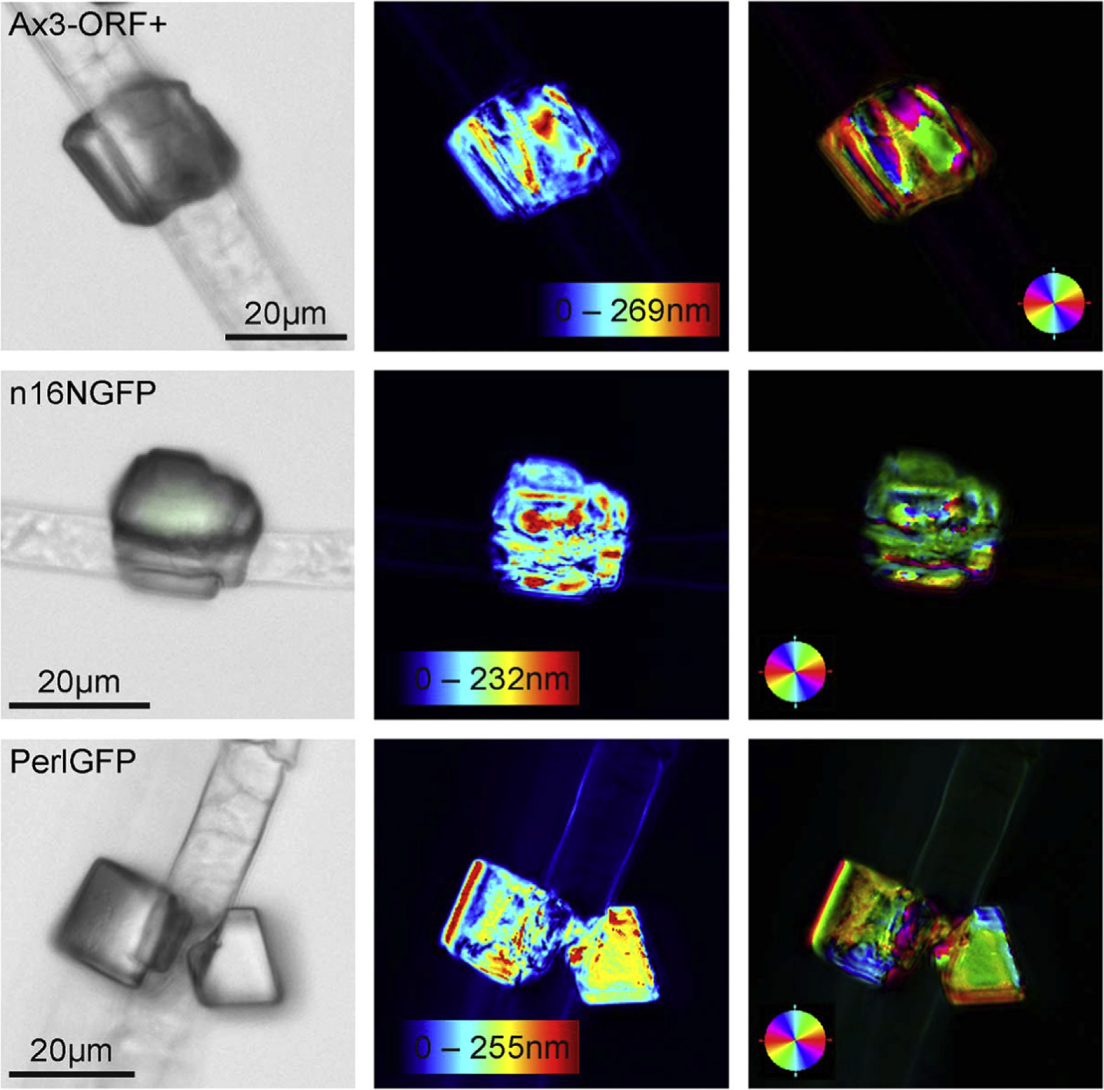
Calcium carbonate precipitates associated with *D. discoideum* Ax3-Orf+ stalks as obtained by the ammonium carbonate vapor diffusion protocol. Region 3, the so-called stalk region, of Ax3-Orf+ (top row), AX3_n16NGFP (central row) and, AX3_PerlGFP (bottom row), in transmitted light and fluorescence overlay microscopy images (left column), and LC-PolScope images analyzed in the retardance mode (center) and in the orientation mode (right column), with slow optical axis indicated in red 0°/180°, light blue 90°/270°.

The birefringence of specimens was analyzed using a LC-PolScope image processing system operated by the Abrio software (CRI, LOT-Oriel, Darmstadt, Germany) [6], [7]. This system was mounted on the inverted light microscope Cell Observer Z1 (Zeiss Observer Z1, Göttingen, Germany). All background images were captured under identical conditions as the area of interest, with the only difference being the absence of the specimen. The measured birefringent retardance is encoded per pixel and displayed in gray scale or false colors. The retardance values represent the relative phase shift between two orthogonally polarized light waves after traversing an optically anisotropic material. Orientation values indicate the azimuth of the slow optical axis (Figs. 2 and 3).

### 2.5. Environmental scanning electron microscopy (ESEM)

**Fig. 4:**
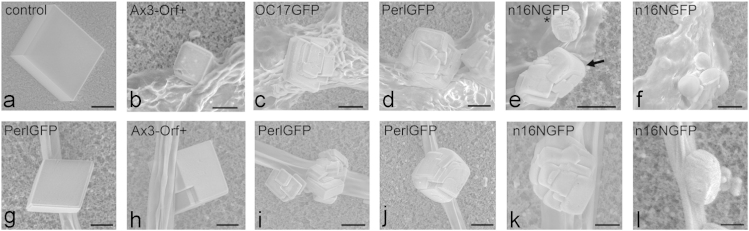
Scanning electron microscopy (VP-SEM) images of calcium carbonate precipitates associated with non-transformed (Ax3-Orf+) and three transformed *D. discoideum* cell lines (AX3_n16NGFP, AX3_OC17GFP, AX3_PerlGFP) obtained according to the slow vapor diffusion protocol. The data show calcium carbonate minerals precipitated from solution without *Dictyostelium* ECM (a), and minerals precipitated in the presence of *Dictyostelium* ECM in region 1 (b–f), and region 3 (g–l). Bars: (a) 20 μm, (b-i, l) 10 μm, (j) 15 μm, (k) 5 μm.

**Fig. 5:**
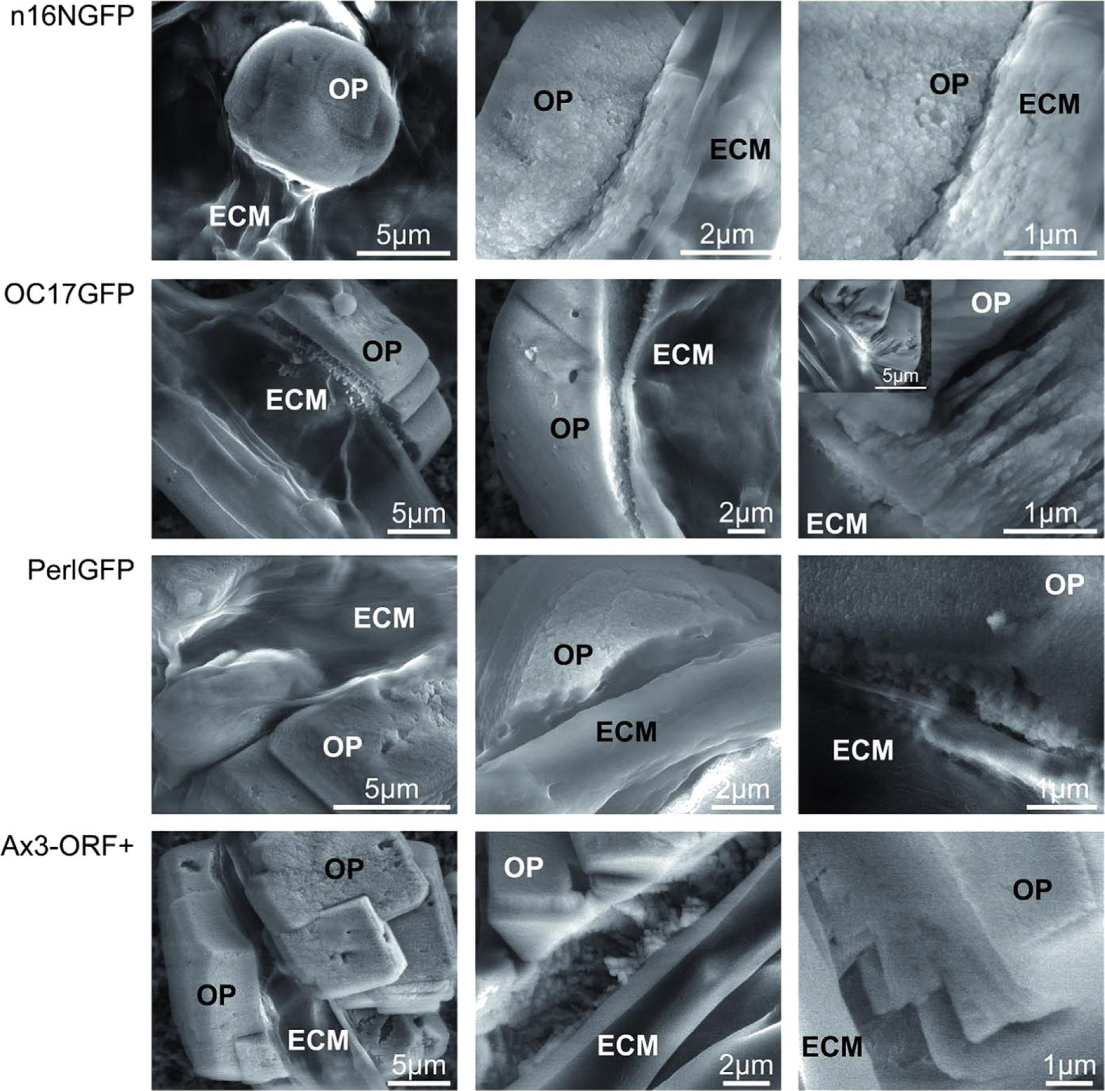
Calcium carbonate precipitates associated with *D. discoideum* Ax3-Orf+ stalks as obtained by the ammonium carbonate vapor diffusion protocol. Low-vacuum scanning electron microscopy (VP-SEM) images of ECM/cellulose stalk-associated mineral precipitates of transformed AX3_n16NGFP (top), AX3_OC17GFP (2nd row) AX3_PerlGFP (3rd row), and non-transformed Ax3-Orf+ (bottom). ECM, extracellular organic matrix; OP, outer mineral precipitate.

Scanning electron microscopy was performed using a Quanta FEI 400F (FEI, Eindhoven, Netherlands) variable pressure SEM (VP-SEM) in either the environmental scanning electron microscopy mode (ESEM) or the low-vacuum SEM mode. Specimens were imaged in native state without additional coating. For ESEM, the stage was cooled to 3 °C before sample application and the chamber was purged 8 times between 800 Pa and 1200 Pa during vacuum pumping. Images were captured with a secondary electron detector at an operating voltage between 10 kV and 30 kV, a spot size of 3, and a pressure of 760 Pa. The low-vacuum mode was commonly used with a secondary electron detector at 10–20 kV and 100 Pa pressure for dry specimens either directly on nitrocellulose membranes or mounted on a silicon wafer (Figs. 4 and 5).

### 2.6. Raman imaging spectroscopy

**Fig. 6:**
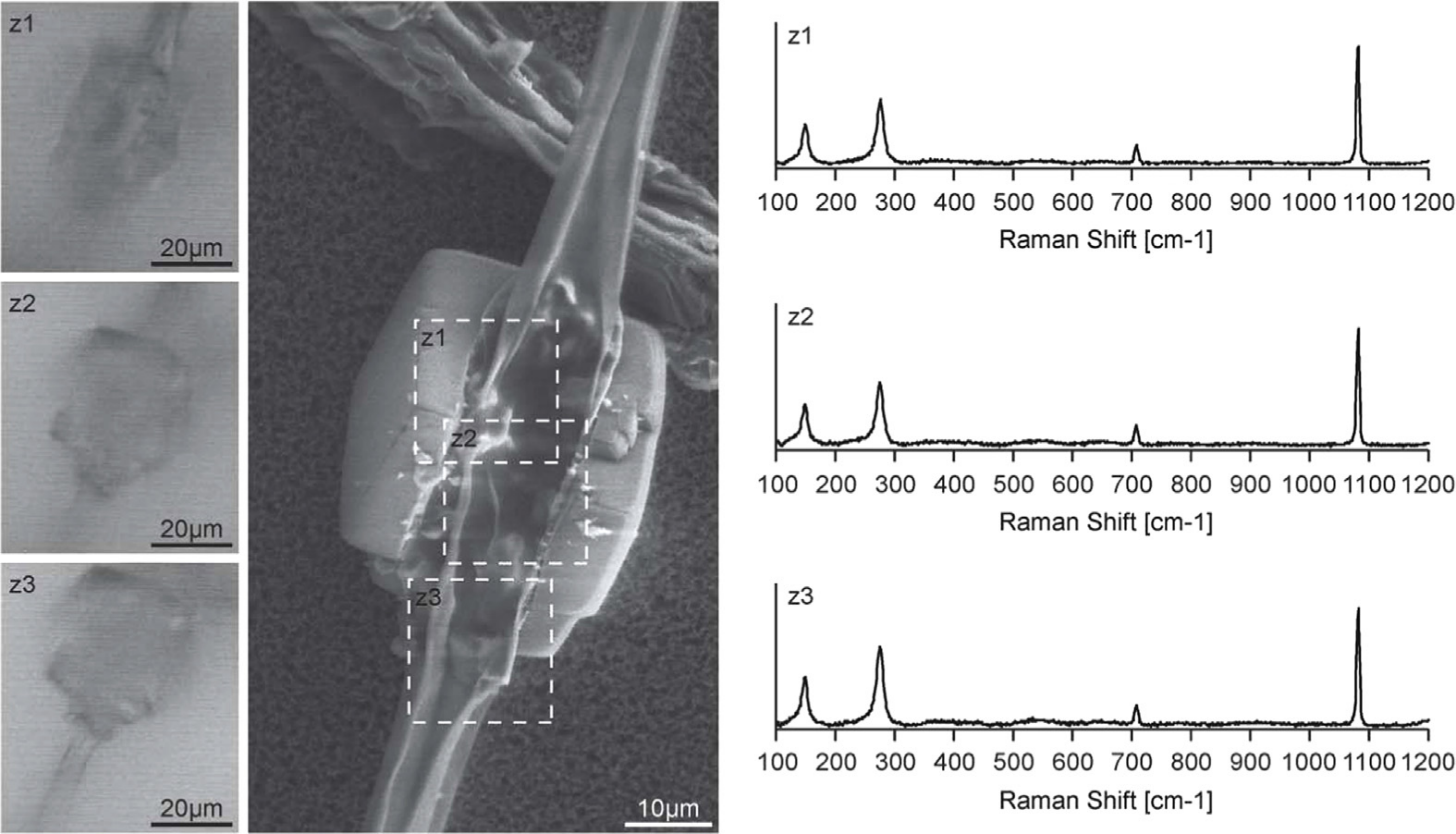
Raman imaging microscopy data (z1–z3) and VP-SEM image of Ax3-Orf+. Raman spectra (right) were taken from various sites (z1, z2, z3) of the crystal surrounding the stalk as indicated in the SEM image (dotted lines).

**Fig. 7:**
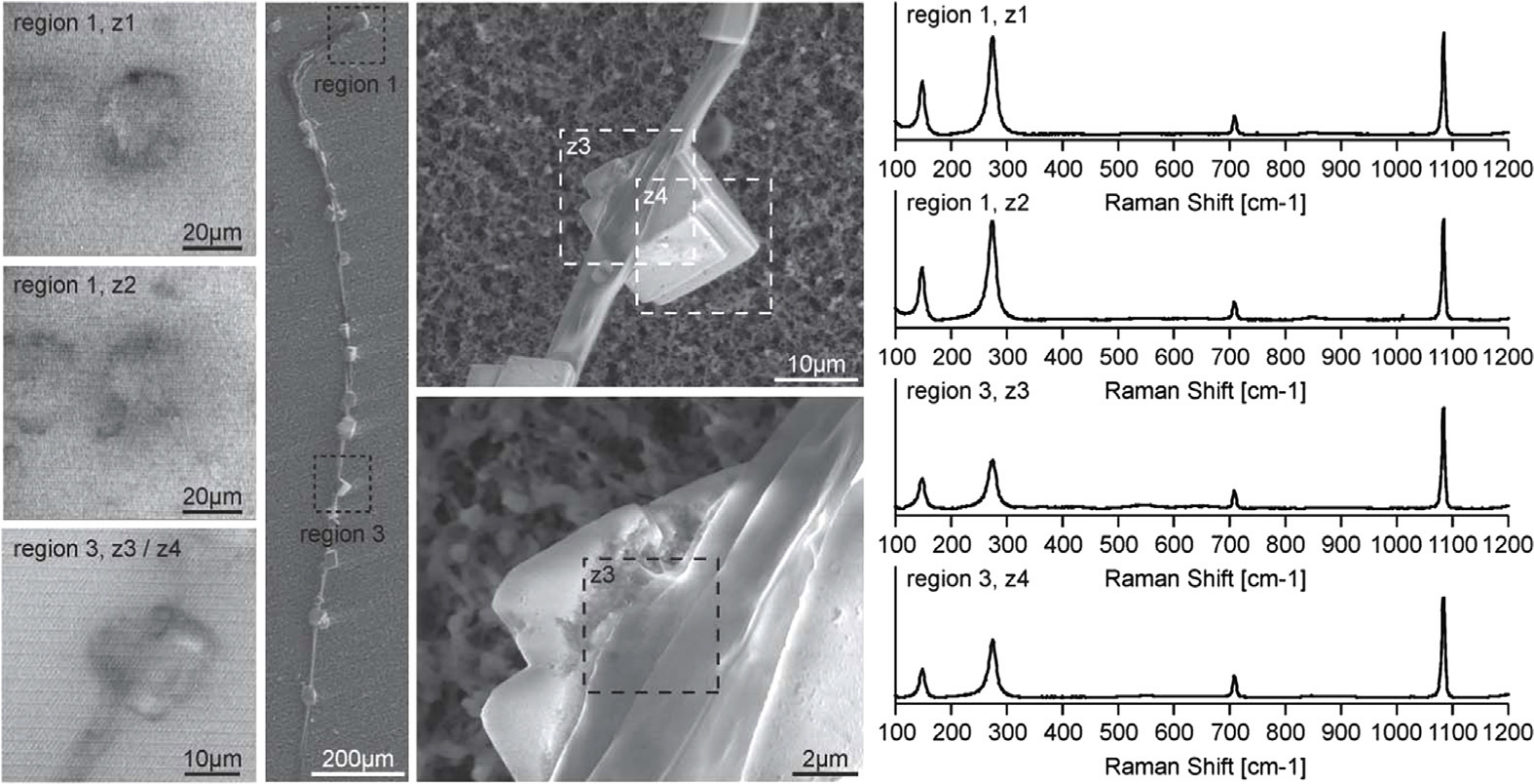
Raman imaging microscopy data (left column) and low-vacuum VP-SEM images (overview, and region 3 of the stalk at higher magnification) of AX3_OC17GFP. Raman spectra (right) of mineral precipitates attached to region 1 (z1, z2) and region 3 (z3, z4) with corresponding images taken with the Raman microscope (left) are shown. A focal series of Raman spectra was collected manually from the mineral precipitate (z1, region 1) and underneath the mineral in the matrix (z2, region 1).

**Fig. 8:**
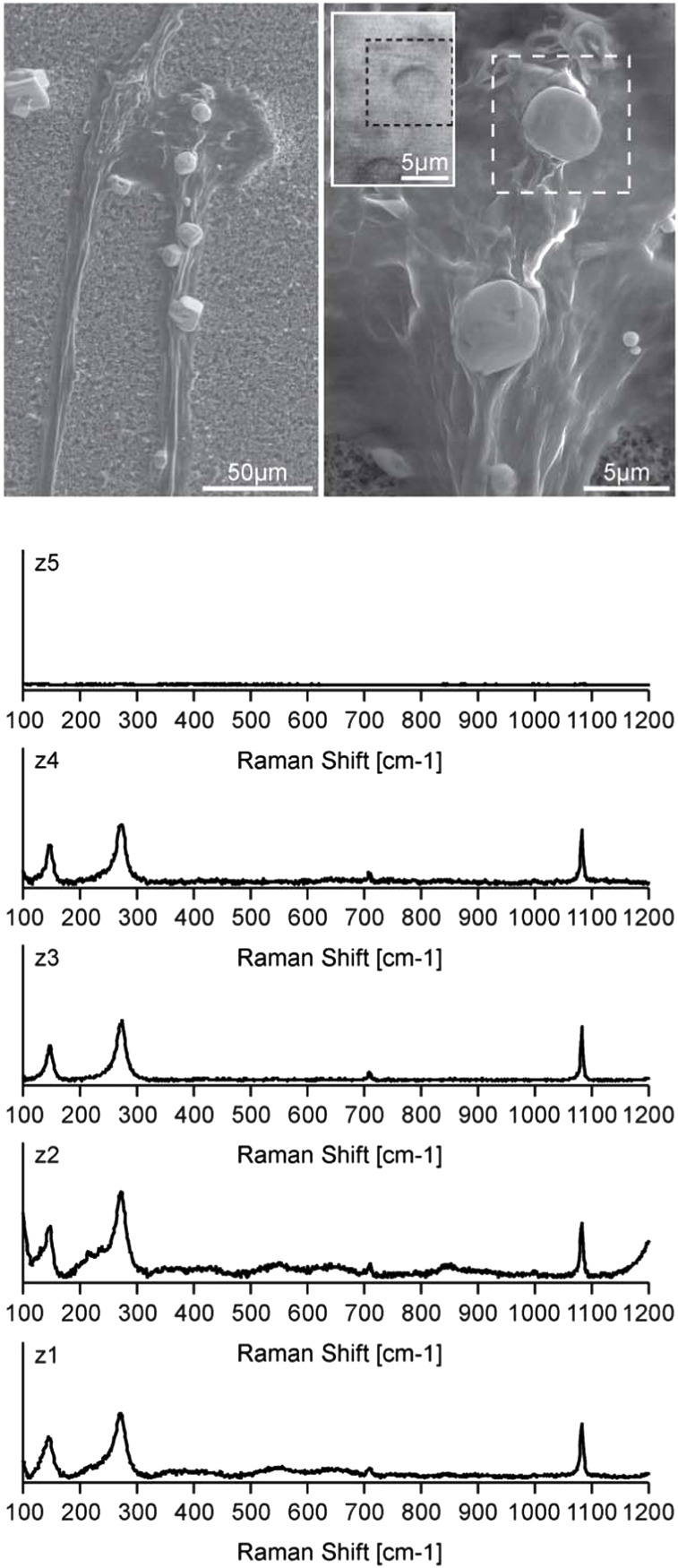
Low-vacuum VP-SEM images (top) and Raman imaging spectroscopy data (bottom) obtained from of a mineral particle precipitated in region 1 of AX3_n16NGFP (top right, dashed lines). The corresponding light microscopy image captured with the Raman microscope is shown in the inlet (top right). Raman spectra were collected in the confocal mode with 5 steps starting in the nitrocellulose filter (z1) through the mineral precipitate (z2 to z4) and ending slightly above the particle (z5). Spectra are adjusted to the height of the 1086 cm^−1^ band.

Raman spectra were collected using a confocal Raman spectrometer (Aramis Labram, Horiba Jobin Yvon, Bensheim, Germany) equipped with an peltier cooled detector. Samples were excited with 785 nm laser and captured using 50×and 100×objectives, a lattice of 600 over a scan range of 100–2000 cm^−1^ and an exposure time of 10 s and 5 integrations. For confocal Raman spectra, the pinhole was closed to 100 μm. For each mineral precipitation experiment, spectra from at least three independent experiments were investigated (Figs. 6-8).
